# Unilateral ossified ligamentum flavum in the high cervical spine causing myelopathy

**DOI:** 10.4103/0019-5413.49385

**Published:** 2009

**Authors:** Udit Singhal, Manoj Jain, Awadhesh K Jaiswal, Sanjay Behari

**Affiliations:** Department of Neurosurgery, Sanjay Gandhi Postgraduate Institute of Medical Sciences, Lucknow - 226 014, India; 1 Department of Pathology, Sanjay Gandhi Postgraduate Institute of Medical Sciences, Lucknow - 226 014, India

**Keywords:** Cervical spine, laminectomy, ossified ligamentum flavum, myelopathy

## Abstract

High cervical ossified ligamentum flavum (OLF) is rare and may cause progressive quadriparesis and respiratory failure. Our two patients had unilateral OLF between C1 and C4 levels. MR showed a unilateral, triangular bony excrescence with low signal and a central, intermediate or high signal on all pulse sequences due to bone marrow within. There was Type I thecal compression (partial deficit of contrast media ring). The first patient had a linear and nodular OLF with calcification within tectorial membrane, C2–3 fusion and unilateral C2-facetal hypertrophy; and the second patient, a lateral, linear OLF with loss of lordosis and C3–6 spondylotic changes. A decompressive laminectomy using “posterior floating and enbloc resection” brought significant relief in myelopathy. Histopathology showed mature bony trabeculae, bone marrow and ligament tissue. The coexisting mobile cervical vertebral segment above and congenitally fused or spondylotic rigid segment below the level of LF may have led to abnormal strain patterns within resulting in its unilateral ossification. In dealing with cervical OLF, carefully preserving facets during laminectomy or laminoplasty helps in maintaining normal cervical spinal curvature.

## INTRODUCTION

The relative incidence of ossified ligamentum flavum (OLF) in the thoracic, lumbar and cervical spine is approximately 38.5% and 26.5% and 0.9%, respectively.^1^–^4^ In the entire cervical spine, 50 cases have been reported till date. 5–11 In the high cervical region (C1–4), however, its occurrence is extremely rare^5^,^7^,^11^ where progressive quadriparesis and respiratory failure may result. Systemic hyperostosis and/or dynamic and static stress on the ligament may be the pathogenetic mechanisms.^1^ We report two cases of unilateral, isolated, nontraumatic, high cervical OLF.

## CASE REPORT

### Case 1

**Figure 1:**
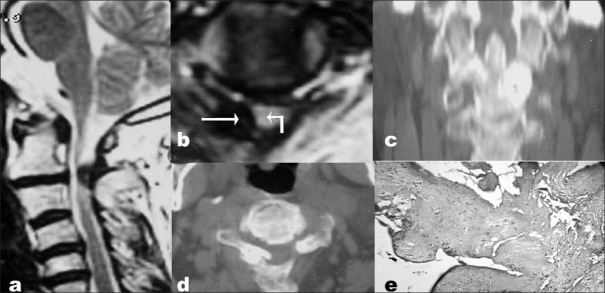
(a) T2-weighted sagittal MRI showing the OLF between the posterior arch of atlas and the fused C2–3 posterior elements causing thecal compression and cord intensity changes. There is an area of hyperintensity within the hypointense rim signifying bone marrow formation. There is also calcification of the tectorial membrane at the level of the tip of the odontoid process and the arch of atlas but without thecal compression. (b) T2-weighted axial MRI showing the right-sided lateral ligamentum flavum ossification (straight arrow) causing thecal compression (curved arrow). The coronal (c) and axial (d) CT images showing the right-sided lateral nodular ossification of ligamentum flavum with hypertrophy of left C2 facet joint. (e) The histopathology of the excised ligamentum flavum revealed bits of mature bony trabeculae, bone marrow and ligament tissue (H&E; x200)

This 50-year-old female patient presented with a 3-year history of asymmetrical spastic quadriparesis (Grade II/III) and hesitancy and precipitancy of micturition. She had hyperreflexia in all 4 limbs, severe spasticity and loss of tactile, pain and posterior column sensations below C4 level. Her lateral cervical radiograph and T1-weighted magnetic resonance imaging (MRI) revealed C2–3 posterior element fusion with the hypointense ligamentum flavum (LF) extending from the lower border of C1 posterior arch to C2-3 posterior elements. The T2-weighted images showed the hypointense lesion with central hyperintensity (suggesting central marrow) causing right-sided thecal compression with cord intensity changes. There was also calcification of the tectorial membrane at the tip of the odontoid process and C1 arch but without thecal compression [Figure 1a and b]. The coronal and axial computed tomographic (CT) scan images revealed a right-sided OLF having linear with central nodular calcification with a central hypodensity due to marrow formation [Figure 1c and d]. The screening MRI of rest of the spine was unremarkable.

A decompressive laminectomy was performed. The fused C2–3 laminae and OLF were drilled in the lateral spinal gutter. The spinal canal was tight with loss of epidural fat at C2–3 level. The OLF was adherent to the outer layer of the dura. It was excised with this layer after it became freely floating on the thecal sac. Thus, the outer layer of the dura was excised, but the inner layer remained intact; therefore, there was no CSF leak. The postoperative course was uneventful. The histopathology revealed mature bony trabeculae, bone marrow and ligament tissue [Figure 1e]. At follow up after 12 months, she had regained normal power in limbs with decreased spasticity.

### Case 2

This 65-year-old male patient had Lhermitte's phenomenon with progressive spastic quadriparesis for 3 months without sphincteric involvement. He had grade IV power in upper and lower limbs, severe spasticity, hyperreflexia in all 4 limbs, absence of superficial abdominal reflex and flexor spasms. The sensory examination revealed hypoesthesia below C5 dermatome with loss of posterior column sensations in upper and lower limbs. His chest expansion was 1.5 cm with shallow respiration.

**Figure 2:**
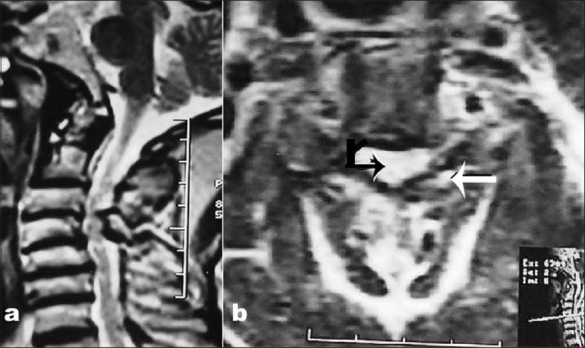
T2-weighted sagittal (a) MRI showing an OLF opposite the C3–4 vertebral bodies. There is associated cervical kyphosis with decreased disc space at the C3–4, C4–5 and C5-6 levels with significant spinal canal compromise and hyperintense cord changes. The axial weighted images (b) reveal a left-sided laterally situated, linear OLF (horizontal straight arrow) causing thecal compression (curved arrow)

His cervical MRI revealed a left-sided OLF causing thecal compression opposite C3–4 vertebrae with a peripheral hypointense rim on T1- and T2-weighted images and central area of hyperintensity on T2-weighted images suggestive of marrow formation. Hyperintense T2 cord signals were present at the level of compression. There was loss of cervical lordosis due to associated spondylotic changes and decreased disc space at C3–6 levels [Figure 2a and b]. A screening MRI of the rest of the spine did not reveal any other abnormality. A C3–C5 laminectomy with excision of the OLF was performed. The LF was drilled in the left lateral spinal gutter after making it wafer-thin. Its inner cortical margin was cut using a micro-Kerrison punch. There was no dural breech. The patient placed on overnight elective ventilation. The postoperative course was uneventful without any complications. At a 3-month follow up, he was able to walk without support and had resumed independence in activities of daily living. His respiratory efforts showed significant improvement.

## DISCUSSION

The uniqueness of our two cases with OLF lies in their being unilateral and high cervical in location. There was no history of systemic hyperostosis or trauma.

OLF frequently occurs at the thoracic and thoracolumbar regions below C6–7, as ossification starts at the densely adherent ligament-osseous junction (enthesis); increased LF craniocaudal thickness combined with a narrower thoracic canal diameter leads to its earlier detection; and static tension causes decreased elastic fibers and the emergence of hyalinized collagen fibers, fibrocartilaginoid cells and interligamentous calcification.^4^,^7^,^8^

Cervical OLF is usually rare as cervical flexion-extension enables LF to frequently becomes lax and thus maintain its elastic fibres.^7^ The presence of cervical OLF in our patients may have been due to changed biomechanical stresses brought about by variations in the osseous configuration of their cervical spine. Case 1 had C2–3 fusion, and Case 2 had spondylotic changes with restricted movement at the C3–6 levels. This may have led to a disproportionate and repetitive stress on the ligament interpositioned longitudinally between the mobile atlas above and the rigid C2–3 vertebral segment below, in Case 1; and between the mobile atlas and axis above, and the relative rigid, spondylotic segment C3–4–5–6, in Case 2. A similar situation in the thoracolumbar region produces stress on the LF between the rigid rib cage above and the flexible lumbar spine leading to a higher frequency of OLF in this region.^4^ Under this cyclical stress, significant degeneration of elastic fibers, fibre bundle degeneration, decrements in fiber diameter and fragmentation occur. The progress of calcification and ossification are closely associated with the degeneration of elastic fibers with expression of bone morphogenetic protein-2, transforming growth factor beta, and vascular endothelial growth factor in the ossification front.^11^

Unilateral cervical OLF has been rarely reported.^5^,^7^,^10^,^11^ This may be a manifestation of ossification initiated at facet joint capsule. Lack of uniformly distributed tension of the ligament between the dynamic segment above and the fused segment below may also be responsible.

In our patients, the characteristic MRI features of OLF were the triangular bony excrescence protruding into the spinal canal that had a low signal on spin-echo or gradient-echo images due to lack of protein in the calcified tissue; with central intermediate or high signal on all pulse sequences and especially on T2-weighted images due to the bone marrow within.^9^,^10^

The appearance of OLF on CT may be of three types: lateral, diffuse and thick, nodular types.^6^ Our first patient had both lateral and nodular, and our second patient had a predominantly lateral type of OLF. Case 1 also had segmental calcification within the tectorial membrane at the level of odontoid, and Case 2 had coexisting subaxial cervical spondylosis. A strong association with ossified posterior longitudinal ligament and with OLF at other sites of the spine has also been noted.^9^ An asymptomatic cervical OLF associated with the symptomatic thoracic one has been reported emphasizing the role of screening MRI of the entire spine.^3^ Both of our patients, however, did not have OLF at multiple sites. An association between facet joint hypertrophy and OLF as seen in our first case may also occur.^9^ Our second case had kyphosis of the cervical spine; this curvature change has also been proposed to have an impact on the genesis of the OLF.^9^

The degree of spinal cord compression by the OLF on intrathecal contrast CT has been classified as: Type I: partial contrast media ring deficit; Type II: narrowing of characteristics of contrast media ring; and Type III: disappearance of contrast media ring.^2^ The greater the grade, less was the predicted neurological recovery. The MR T2-myelographic effect showed that both our patients had Type I compression due to the unilateral OLF. As predicted by the classification, there was significant neurological recovery in our patients at follow up.

A decompressive laminectomy including one level above and below the lesion was the procedure of choice.^4^,^11^ A medial facetectomy was avoided since cervical spine, in comparison to thoracic spine (stabilized by rib cage), is prone to developing kyphosis and instability even with 25% facetectomy.^13^ During drilling, cord injury was avoided by gently making the OLF wafer-thin; disconnecting it from the vertebral body in the lateral spinal gutters on the side of the dura using micro-Kerrison's punches; and then gently elevating it from the dura (the posterior floating and en bloc resection technique). In case the dura is densely adherent to the OLF and gets opened during its excision, then a fat fascial graft may be placed with fibrin glue in the gutter created. In case the rent is large, then a lumbar cerebrospinal fluid drain for three days with oral acetazolamide 250 mg QID may be added. In our first case, the OLF was adherent to the outer wall of the dura and had to be excised along with it. A pure foraminotomy and extended hemilaminectomy would have caused difficulty in defining the OLF-duramater interface and also in controlling epidural bleeding by the narrow surgical corridor. Extending the laminectomy above and below the level of the compression helped in defining the dural level before approaching the site of maximum compression. Since facet joints were not compromised, posterior lateral mass instrumentation was not required. Our second patient had loss of normal cervical lordosis due to the coexisting spondylosis. This patient may develop a swan-neck deformity due to the laminectomy and is on regular follow up with serial imaging.^12^ In this case, either a laminoplasty with preservation of posterior bony vertebral structures or laminectomy and instrumented fusion would be alternatives to the laminectomy that was performed.^3^

To conclude, two rare cases of focal, isolated, unilateral cervical OLF are presented. The coexisting mobile vertebral segment above and the congenitally fused or spondylotic rigid segment below may have led to abnormal strain patterns on LF that caused unilateral ossification. In dealing with cervical OLF, carefully preserving facets during laminectomy helps in maintaining normal cervical spinal curvature.
